# Supplementary material for: Training spiking neuronal networks to perform motor control using reinforcement and evolutionary learning
Source: Front Comput Neurosci. 2022 Sep 30;16:1017284. doi: 10.3389/fncom.2022.1017284 (PMC9563231; doi:10.3389/fncom.2022.1017284)
Supplement: Supplementary file 1 [file Data_Sheet_1.docx]

**Supplementary Figures**

~~
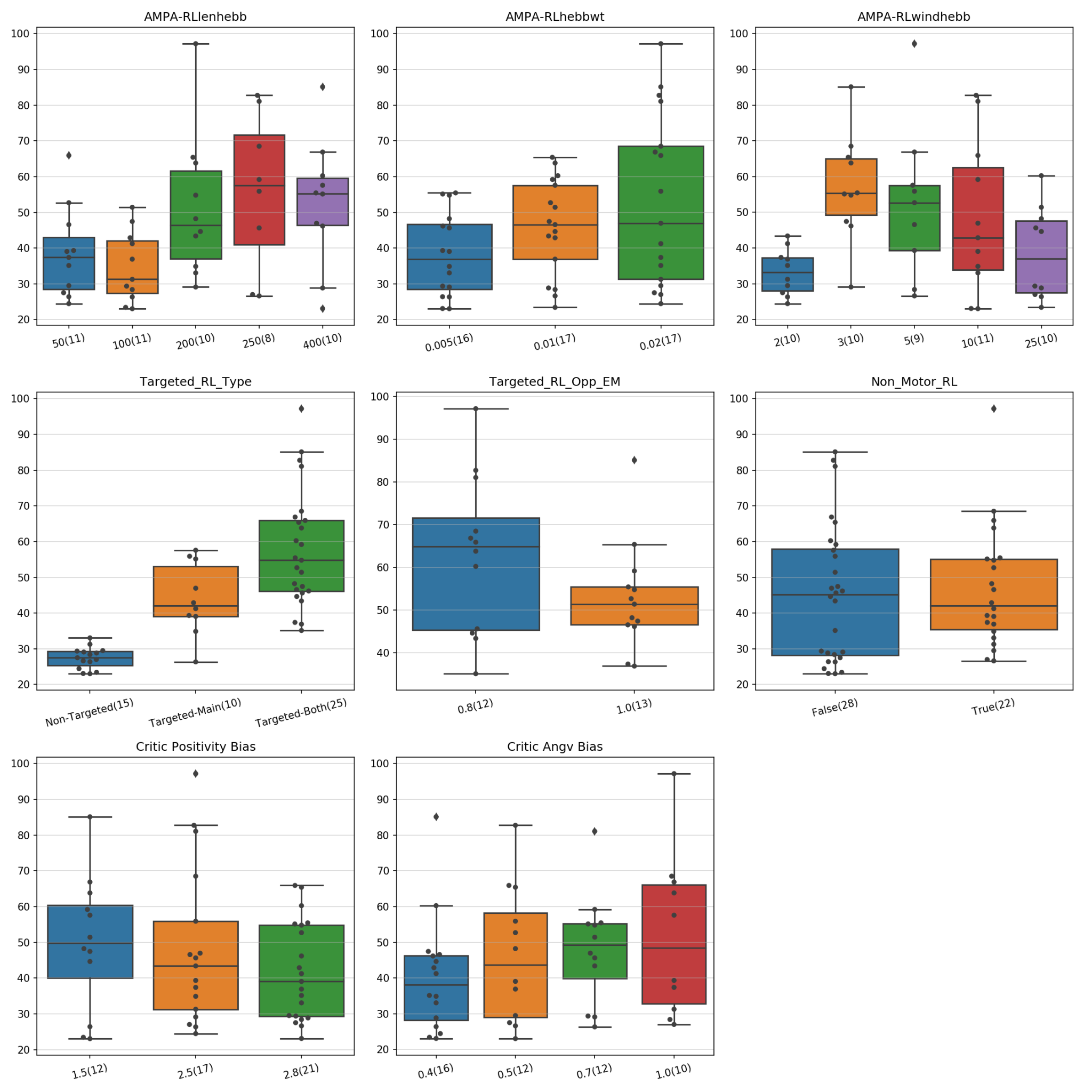
~~

***Supplementary Figure 1:*** ***Performance distribution of the first hyperparameter search for training using STDP-RL (displaying averages over 100 episodes during training).*** *In each panel, the y-axis shows the performance, and the x-axis indicates the parameter values used in the evaluation. The number of models run with the specific parameter value is presented in parentheses, with 107 tested hyperparameter combinations.*


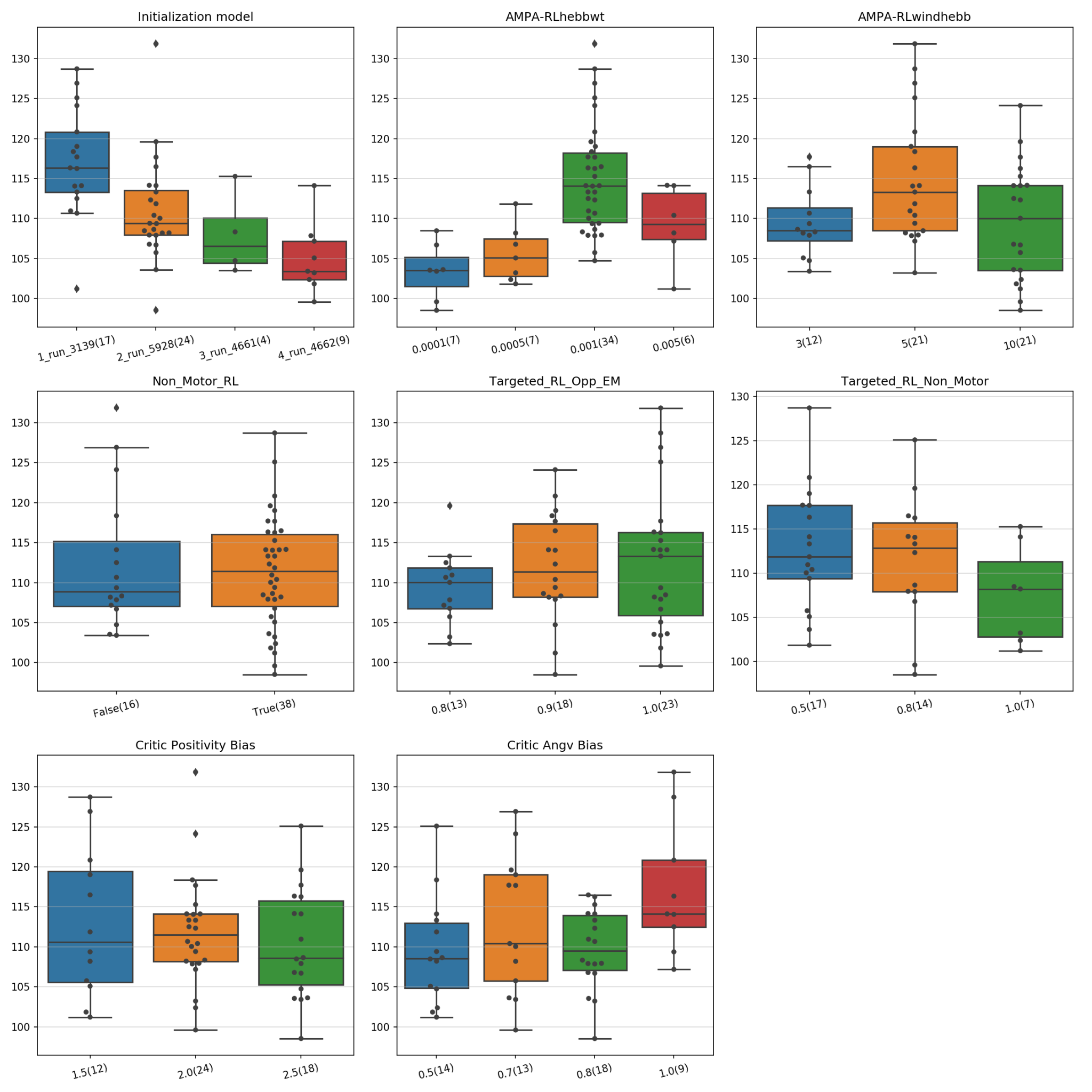


***Supplementary Figure 2:*** *Performance distribution of hyperparameters for training using STDP-RL evaluated in the second step (using averages over 100 episodes).*


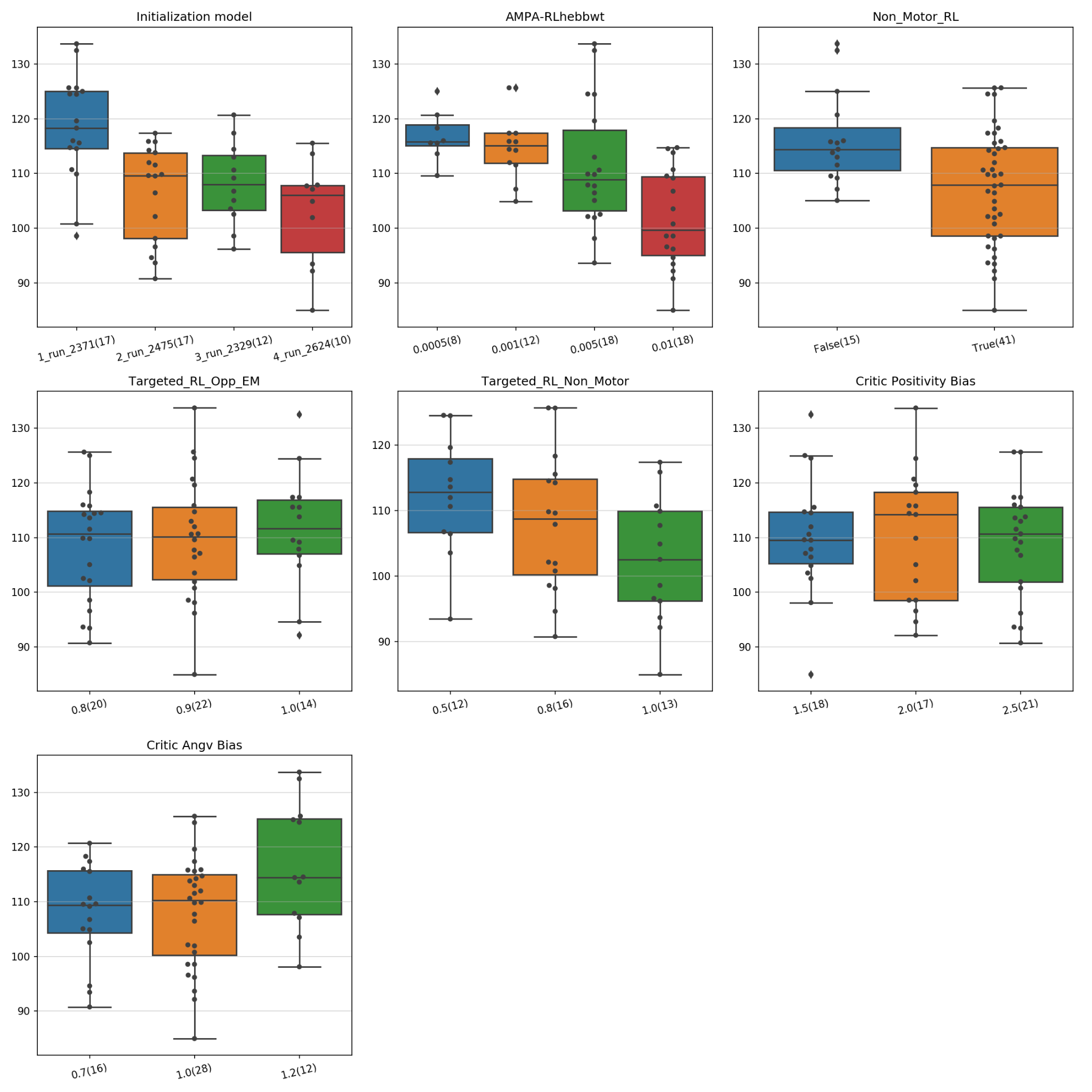


***Supplementary Figure 3:*** *Performance distribution of hyperparameters for training using STDP-RL evaluated in the third step (using averages over 100 episodes).*


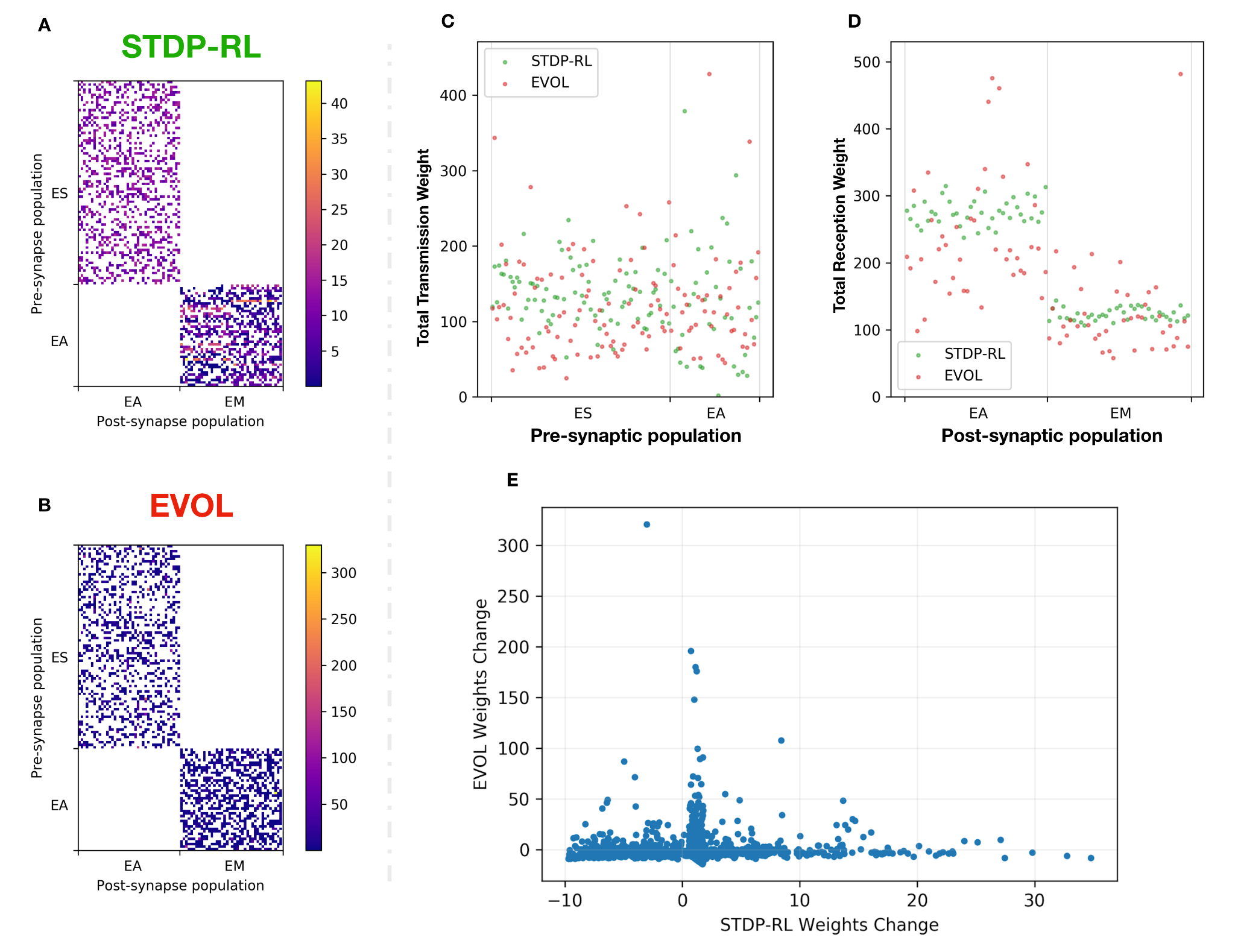


***Supplementary Figure 4: STDP-RL and EVOL differentially modulate the synaptic weights of the SNN model during training. (A-B)*** *Adjacency matrices showing the weights of synaptic connections between ES-EA and EA-EM populations in STDP-RL model* ***(A)*** *and EVOL model* ***(B).*** *Note that the adjacency matrices have different scales as the EVOL model has weights reaching 300.* ***(C)*** *Total Transmission Weight: Sum of synaptic weights from a presynaptic neuron onto multiple postsynaptic neurons.* ***(D)*** *Total Reception Weight: Sum of synaptic weights onto each postsynaptic neuron from multiple presynaptic neurons.* ***(E)*** *As each model changed the weights from the original initialization, each dot represents a synaptic connection weight after training with STDP-RL (x-axis) and EVOL (y-axis) with a Spearman correlation of -0.09 (p-value = 0.0001).*

***Supplementary Table 1:*** ***STDP-RL parameters obtained through hyperparameter search.*** *Values tested that are bolded are the hyperparameters picked for our model.*

| **Parameter** | **Hyperparameter Search Step 1** | **Hyperparameter**  **Search Step 2** | **Hyperparameter**  **Search Step 3** | **Description** |
| --- | --- | --- | --- | --- |
| Duration (s)  (not varying) | **500** | **2000** | **2000+** | Duration in seconds that was trained using each. A step was performed in 50ms |
| AMPA-RLwindhebb (ms) | (2, **3**, 5, 10, 25) | (3, **5**, 10) | **5** | Maximum time between presynaptic and postsynaptic spike times for considering plasticity. |
| AMPA-RLlenhebb (ms) | (50, 100, 200, 250, **400**) | **250** | **250** | The decay time constant of the exponentially decreasing eligibility trace. |
| AMPA-RLhebbwt | (0.005, 0.01, **0.02**) | (0.0001, 0.0005, **0.001**, 0.005) | (0.0005, 0.001, **0.005**, 0.01) | Max synaptic weight adjustments based on reward or punishing signal. |
| Targeted_RL_Type | (non-targeted RL,  targeted RL main,  **targeted RL both**) | **targeted RL both** | **targeted RL both** | The Targeted RL paradigm chosen |
| Non_Motor_RL | (**False**, True) | (**False**, True) | (**False**, True) | Activating STDP-RL plasticity for synapses in the $ES\to EA$ pathway |
| Targeted_RL_Opp_EM | (0.8, **1.0**) | (0.8, 0.9, **1.0**) | (0.8, **0.9**, 1.0) | Attenuation factor for the opposite subpopulation receiving reinforcement |
| Targeted_RL_Non_Motor | **1.0** | (0.5, 0.8, **1.0**) | (0.5, 0.8, **1.0**) | Attenuation factor for the non-motor subpopulation receiving reinforcement |
| Critic Positivity Bias | (**1.5**, 2.5, 2.8) | (1.5, **2.0**, 2.5) | (1.5, **2.0**, 2.5) | $\eta_{angvel}$: used in critic (**equation 1**) |
| Critic Angv Bias | (**0.4**, 0.5, 0.7, 1.0) | (0.5, 0.7, 0.8, **1.0**) | (0.7, 1.0, **1.2**) | $\eta_{positivity}$: defined for critic evaluation (**equations 2-3**) |
